# Overexpression of AGT promotes bronchopulmonary dysplasis via the JAK/STAT signal pathway

**DOI:** 10.18632/oncotarget.21712

**Published:** 2017-10-10

**Authors:** Lili Shen, Tiancheng Zhang, Hongyan Lu

**Affiliations:** ^1^ Department of Pediatrics, The Affiliated Hospital of Jiangsu University, Zhenjiang, China; ^2^ Department of Pediatrics, Suzhou Kowloon Hospital Shanghai Jiao Tong University School of Medicine, Suzhou, China; ^3^ Institute of Reproduction and Development, Fudan University, Shanghai, China; ^4^ China National Population and Family Planning Key Laboratory of Contraceptive Drugs and Devices, Shanghai Institute of Planned Parenthood Research (SIPPR), Shanghai, China

**Keywords:** differentially expressed gene, functional enrichment analysis, broncopulmonary dysplasia, angiotensinogen, inflammation

## Abstract

Angiotensinogen (AGT) is involved in the production of angiotensin II which is the main mediator of action of the rennin-angiotensin system (RAS), whereas the RAS mediates the regulation of sodium homeostasis, blood pressure, and inflammation. The present study aimed to investigate the roles of the AGT in the progression of broncopulmonary dysplasia in premature newborns. By bioinformatics analysis, AGT was found to be the major node in molecular interaction networks of BPD mouse model. Quantitative PCR and western blot analyses were applied to examine AGT expression in A549 cells which were treated with the hyperoxic condition. The AGT inhibitor Valsartan and the AGT agonist ANGII were employed to investigate the roles of AGT in cell growth and the inflammation. Results show that hyperoxic treatment induced upregulation of AGT expression in A549 cells. Overexpression of AGT resulted in the inflammation via the JAK/STAT signal pathway, ultimately suppressed the proliferation of the A549 cell. In conclusion, increased expression of AGT was demonstrated to be associated with the development and progression of BPD, and may be regarded as a promising therapeutic target for BPD.

## INTRODUCTION

Bronchopulmonary dysplasiais, a leading cause for many pulmonary diseases in early infancy, was histologically characterized by intense airway inflammation and lung fibrosis, even more it might lead to an increased risk for brain injury [1], severe metabolic bone disease [2]. More and more BPD patients were benefited from the different approaches derived from perinatal care, such as the antenatal corticosteroids treatment, postnatal surfactant therapy, and different ventilation modes et al., but it was found that no obvious improvement in decreasing the incidence of BPD in the last decade and no definite therapy can eliminate this complication. To date, a considerable number of literatures on the BPD were reported to elucidate its pathogenesis and try to find an effective treatment to prevent and/or cure this disease. Our previous studies demonstrated that the hypoxia-induced BPD originated from persistent ER(endoplasmic reticulum) stress, and finally led to upregulation of CHOP expression and cell death [3], Wagenaar *et al* observed that a missense mutation in cytoplasmic helix 8 of LPAR1(lysophosphatidic acid receptor 1) can protect the lung of neonatal rats against the hyperoxia-induced BPD, moreover, treatment of Ki16425 toward BPD experimental mode confirmed this result that blocking of LPAR1 may be a novel therapeutic option for BPD [4]. It was also reported that treatment of hyperoxia-exposed mice with either IL1 receptor antagonist to block IL1 β or glybruide to block NIrp3 inflammasome resulted in decreased inflammation and increased alveolarization, it showed that the early activation of the NLRP3 inflammasome maybe a key mechanism in the development of BPD [5]. Recently, a considerable number of new factors were identified to be associated with bronchopulmonary dysplasia (BPD). For example, breastmilk feeding for the premature infants was identified to be tightly involving in a reduced risk of BPD by meta analysis on about 460 infants [6]. The early anemia was proved to be related with an increased risk of BPD by meta analysis on the 243 infants with gestational age less than 32 weeks [7]. It was found that the number of transfusions was one of the key risk factors for BPD [7], other factors such as virus infection [8], nutrition [9], genetic predisposition [10] were also identified to be associated with the BPD. In all, the great progresses in elucidating the mechanism of the BPD were obtained, but completely understanding to the pathogenesis of the BPD is still in trouble due to the complexity and multifactor in aetiology.

Angiotensinogen (AGT) is involved in the production of angiotensin II which is the main mediator of action of the rennin-angiotensin system (RAS) [11] whereas the RAS mediates the regulation of sodium homeostasis, blood pressure, and inflammation [12], so it was speculated that AGT gene polymorphism might be the key biomarker predicting the risk of HSP/HSPN through its influence on the level of AGT [13]. It was found that the functions of AGT was markedly related to the initiation and progression of cancer [14], due to that the transgenic mice with high levels of AGT are found to be at lower risk of developing cancer [15]. Either high or low levels of AGT have been observed in various human tumor tissues [16, 17]. Moreover previous studies reported that AGT could interact with TGF-β1 to promote fibrogenesis of Lung [18], in addition, there were also reports on the possible association between angiotensin-converting enzyme and angiotensin type I receptor polymorphisms and the risk of the developing BPD [19], but little attention has been directed to the AGT mediated physiologic pathways which is involved in the development of BPD.

In this study, we performed mRNA profiling at postnatal day 14 of murine model to compare hyperoxia-induced bronchopulmonary dysplasia and wild type as the control. Further analysis were performed by combining functional enrichment analysis and interaction network analysis to screen the key genes that were tightly associated with the pathogenesis of BPD. Then different experimental approaches were employed to verify and elucidate the functions of key genes on the BPD.

## RESULTS

### Differential gene expression of murine model between the hyperoxia-induced bronchopulmonary dysplasia and the healthy control

**Figure 1:**
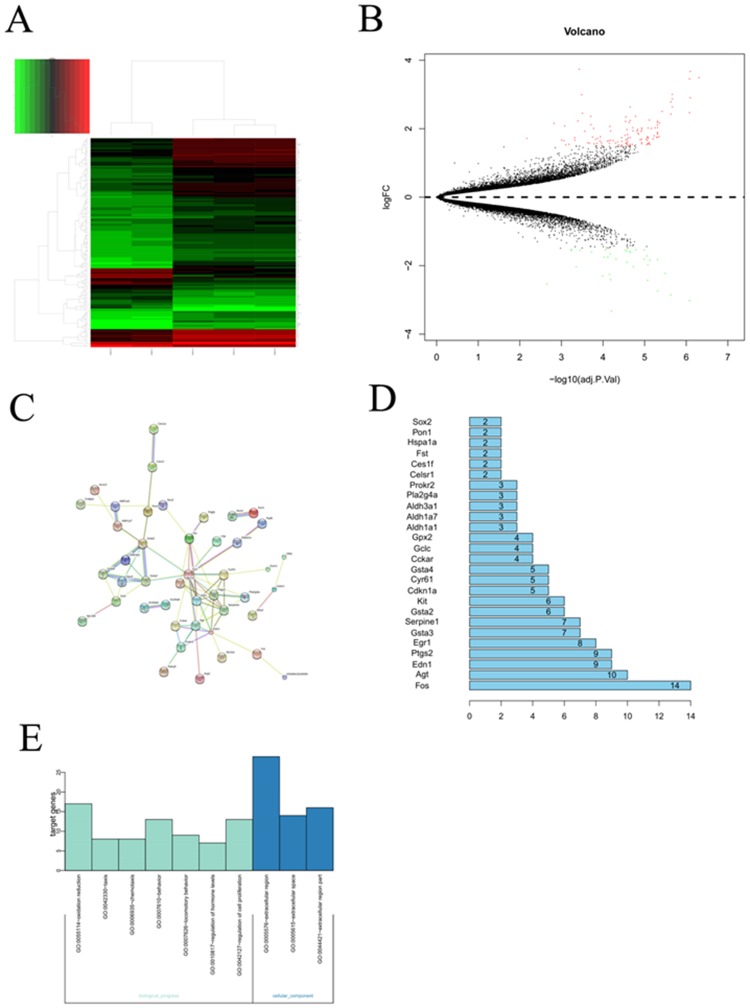
Bioinformatic analysis on the microarray data set GSE25286 **(A)** Cluster analysis for gene expression data, the expression values clustered in the red shaded areas indicated overexpression, and the green-shaded areas indicate underexpression. **(B)** The DEGs in BPD samples compared with those in normal samples. Red dots represented differentially expressed genes, black dots were non-differentially expressed genes. **(C)** Interaction network constructed by target genes of differentially expressed miRNAs. Circles of different colors stand for different genes. Blue lines link two related genes. **(D)** The number of interaction of a protein in the network. **(E)** GO analysis to categorize the differentially expressed genes into GO categories.

To identify the DEGs in murine model with the BPD induced by the hyperoxia compared with the healthy control, the microarray data was analyzed and chi-square test was used for multiple comparisons. As shown in the Figure 1, A total of 134 DEGs were screened out in BPD samples compared with the healthy controls, including 40 upregulated DEGs and 93 downregulated DEGs.

### Protein interaction network analysis of the DEGs

Protein-protein interaction networks were constructed for DEGs from the BPD samples and the healthy control using the STRING and significant cut-off point of combined score >0.4. the network contained 44 nodes and 148 edges, and it was found that the AGT gene can interact with more than 12 genes and also the AGT gene located in the center of the topology network.

### GO analysis on the DEGs

The differentially expressed genes were functionally annotated based on gene ontology (GO). The results showed that the differentially expressed genes were associated with cellular components, biological progress. The GO category analysis revealed that the differentially expressed genes fell into the following categories: regulation of cell proliferation, regulation of hormone, locomotory behavior, oxidation reduction and so on.

### Hyperoxic condition induce the overexpression of the Agt in A549 cell

**Figure 2:**
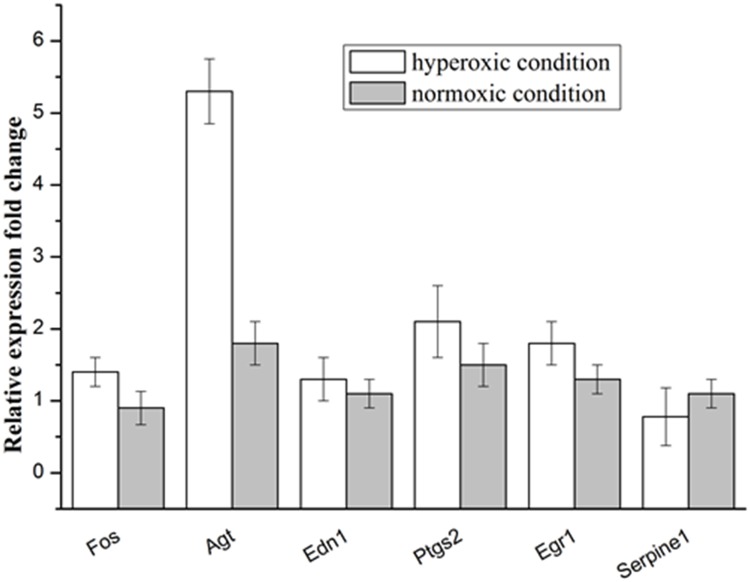
Relative expression levels of selected genes in the A549 cells under the hyperoxic and normoxic conditions

RT-qPCR were applied to evaluate the expression of the selected genes in the A549 under hyperoxic condition, the obtained results were shown in the Figure 2, the expression of the Agt gene was significantly larger in the hyperoxic condition, as compared with that in the normoxic condition. Whereas for the other selected genes, no significant change in expression was observed between in the hyperoxic and normoxic condition. This results showed that hyperoxia could improve the expression of the angiotensinogen (AGT) in the A549 cell.

### AGT overexpression increases markers of lung fibroblasts inflammation

**Figure 3:**
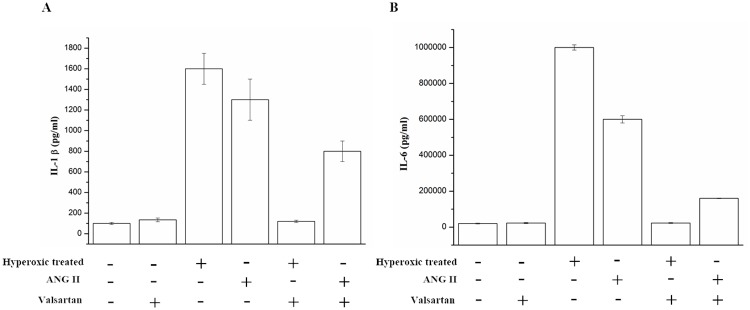
The IL-1β and IL-6 in the supernatant of the A549 cells which were exposed to hyperoxic (Hyperoxic treated), room air(normoxic control) conditions and/or combination with the treatment of adding ANG II or Valsartan, were analyzed by Elisa, Data are expressed as mean±S.D. of three independent experiments

Inflammation was always considered as one of the key factors that resulting in BPD of the infants. We measured selected markers of inflammation in A549 cells which were in the hyperoxic condition for 60h. the proinflammatory cytokine IL-1β[20] were nearly sixteen-fold higher in the hyperoxic treated groups compared to nomoxic controls. While, the expression of interleukin-6(IL-6) was also significantly higher in hyperoxic treated groups, the corresponding measured value reached to almost 10^6^ pg/ml, whereas the control was 3000 pg/ml. These findings indicated that overexpression of AGT can induce inflammation of the A549 cells. Additionally, it was observed that adding ANG II, an peptide derived from the angiotensin, significantly increased the expression of the IL-1β and IL-6, but adding the Valsartan, the angiotensin type I receptor blocker, reduced the overexpression of the IL-1β and IL-6 which derived from the hyperoxic treated and/or ANG II adding (Figure 3). These results indicated that overexpression of AGT induces inflammation of the A549 via the angiotensin type I receptor mediating signal pathway.

### AGT gene silence decreases markers of lung firboblasts inflammation induced by hyperoxic

**Figure 4:**
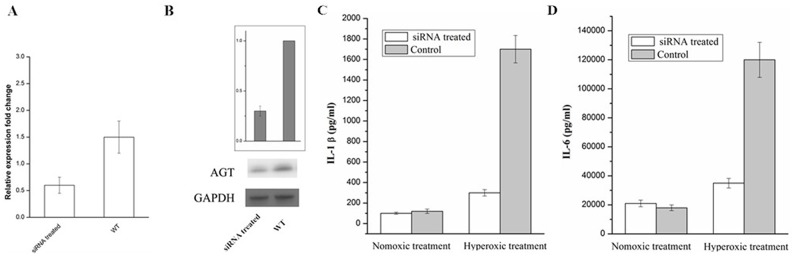
RT-qPCR **(A)** and Western blot **(B)** analysis of AGT expression in A549 cell treated with siRNA and the Control. The IL-1β **(C)** and IL-6 **(D)** in the supernatant of the A549 cells (siRNA treated and NC) which were exposed to hyperoxic (Hyperoxic treated), room air (normoxic control) conditions

Based on the above findings, lipofectamine 2000 was used to transfect siRNA in order to knockdown AGT in A549 cells. The results were shown in the Figure 4A and 4B, the expression levels of mRNA and protein of AGT were significantly decreased under the treatment of the siRNA targeting AGT, indicating that a successful knockdown of AGT was confirmed. Furthermore, the IL-1β and the IL-6 were detected in the supernatant of the A549 cells (siRNA treated and NC) with the treatment of hyperoxic. The obtained results (Figure 4C and 4D) showed that AGT down-regulation decreases markers of lung firboblasts inflammation induced by hyperoxic.

### Overexpression of AGT regulates Jak2/Stat3 signaling pathway

**Figure 5:**
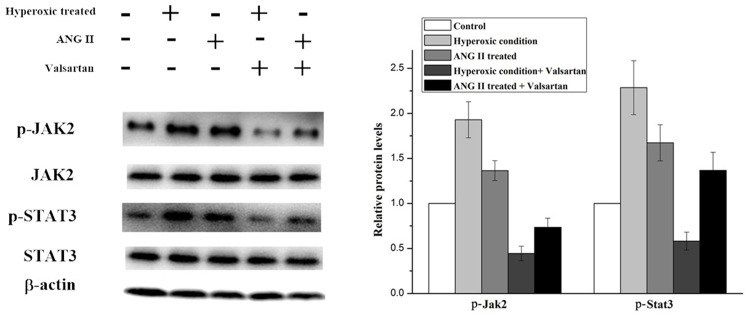
Western blot analysis on the p-Jak2 and p-Stat3 expression level in A549 cells with different treatments

**Figure 6:**
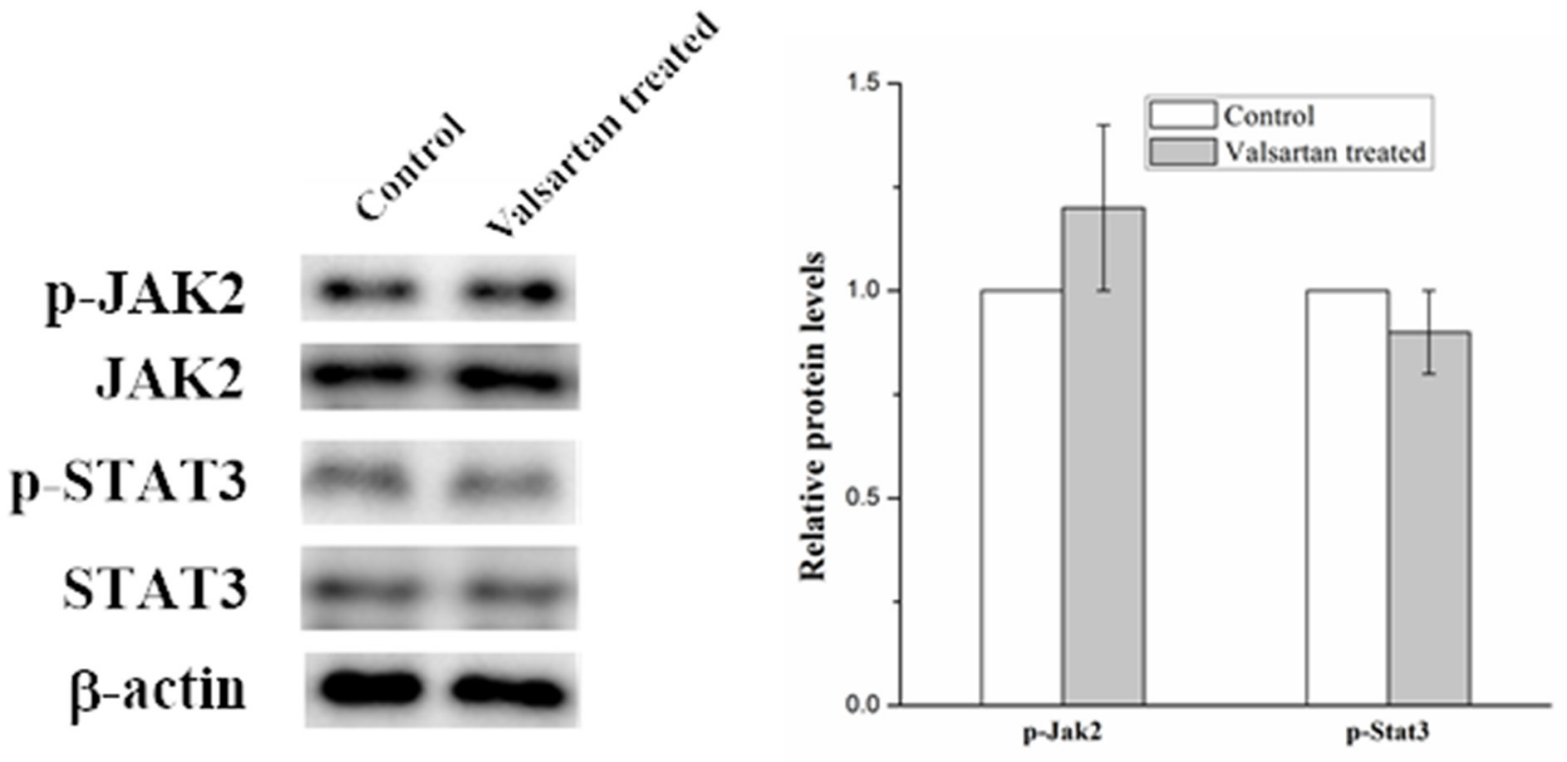
Western blot analysis on the p-Jak2 and p-Stat3 expression level in A549 cells with the treatment of Valsartan

In order to explore the molecular mechanism of inflammation mediated by the overexpression of AGT gene. Several signal pathways which were reported to be associated with the inflammation were investigated, but only the JAK/STAT signal pathway was found to be influenced by the upregulated AGT gene. As shown in Figure 5, the expression of phosphorylated Jak2 and phosphorylated Stat3 were significantly increased in the A549 cell under the hyperoxic condition, the relative expression level of p-Jak2 and p-Stat3 in hyperoxic condition was almost twice than those in the normoxic condition. Whereas adding ANG II play similar roles for A549 cells in elevating the expression of the p-Jak2 and p-Stat3, the p-Jak2 and p-Stat3 were more than the control but less than the hyperoxic treated samples. Moreover, it was found that Valsartan adding can significantly reduce the phosphorylation of the Jak2 and Stat3 caused by hyperoxic treated and ANG II adding, but the protein expression of the Jak2 and Stat3 were no obvious change. In comparison, the Valsartan almost has no effect on the phosphorylation of the Jak2 and Stat3 of the A549 cells which were without the treatment of hyperoxic (Figure 6). These results showed that Hyperoxic treatment increased the expression of AGT gene, the overexpression of AGT promote the activation of the JAK2 and subsequently activated the phosphorylation of Stat3. JAK/STAT, which is the important part of the cell signal pathway and plays a key role in inflammation reaction.

### Overexpression of AGT inhibits the proliferation of the A549 cells

**Figure 7:**
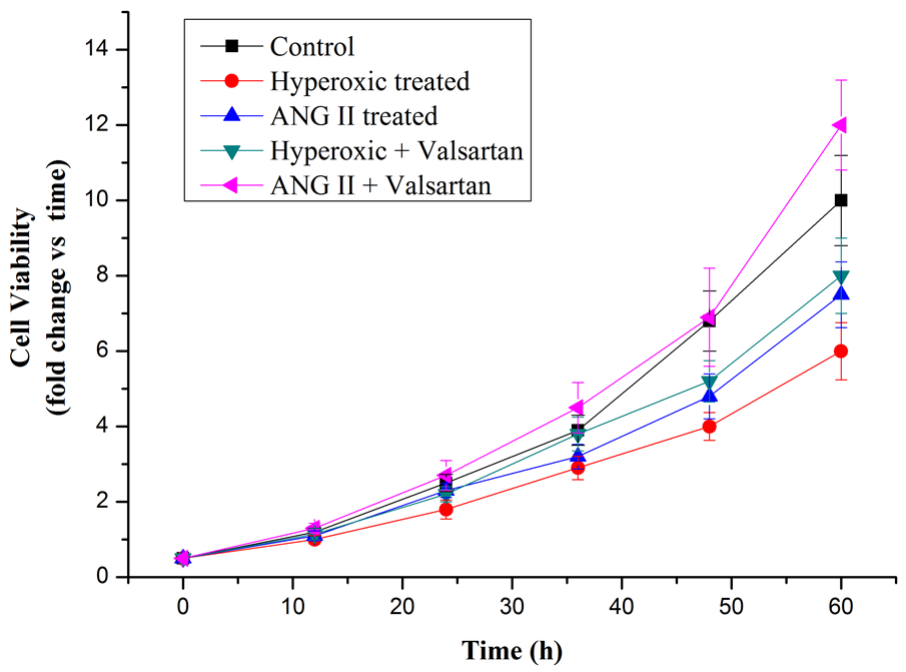
Cell proliferation rate of A549 cells in normoxic condition and in hyperoxic condition as measured by MTT assay

Cell proliferation arrest was the key character of the BPD. To investigate the role of overexpression of AGT on cell proliferation is necessary. Hyperoxic treatment strongly inhibited cell proliferation, as well as the adding ANG II treatment (Figure 7). In contrast, Valsartan, as the blocker of the angiotensin type 1 receptor, significantly restored the cell proliferation which originally inhibited by the hyperoxic treatment, moreover, it was found that with the adding of the Valsartan the cell proliferation of the A549 cells pretreated with ANG II were better than those of the control in 60h. These obtained results indicated that overexpression of AGT gene originated from Hyperoxic treatment suppresses the proliferation of the A549 cells, and the roles of overexpression Agt on the cell proliferation inhibition is dependent on the AGT related signal pathway.

## DISCUSSION

Alveolar development is a complex process involving multiple mechanisms relating to cell cycle, cell adhesion, mobility and taxis, and angiogenesis. BPD is characterized by an arrest in alveolar development and increased apoptosis of alveolar epithelial cells. The inflammation is widely considered as one of the key factors that contribute to lung disease in preterm infants in clinical, because it was observed that ventilator mediated lung injury is able to cause inflammation, and exposure of lungs to high concentrations of oxygen causes severe inflammation. In addition, the experimental literature clearly shows that prolonged ventilation of the developing lung will cause BPD-type anatomical changes associated with persistent inflammation.

**Figure 8:**
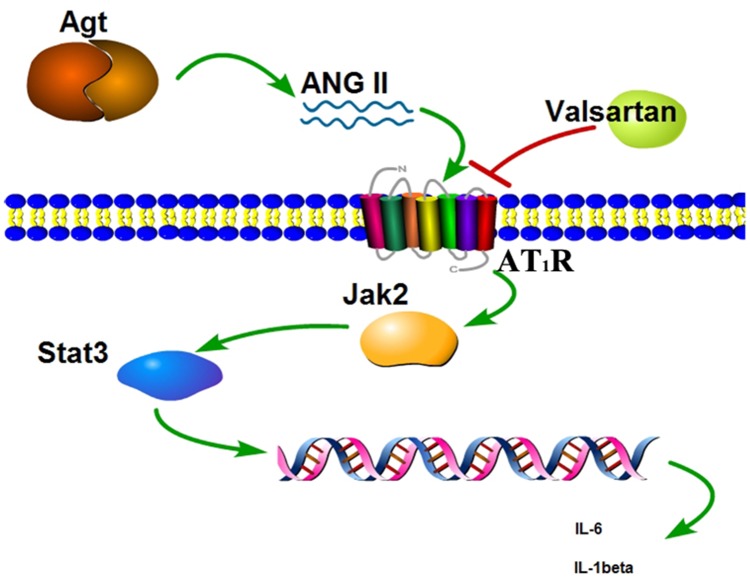
Scheme of the Agt overexpression promote the inflammation factors release via the Jak/Stat signal pathway

Jak2/Stat3 signaling pathway is crucial in numerous signal transductions in cells. The JAKs activation has an important role in cell differentiation, proliferation, apoptosis and migration. Structure activation of JAKs contributes to phosphorylation of the STAT family [21]. STAT3 is a member of STAT family, which has been intensively studied in recent years [22]. As confirmed by cell culture and animal models, over activation of STAT3 is found in various cells with inflammation [23]. Guo et al. have reported that suppressing the activation of the JAK/STAT pathway exerts a inflammation inhibition ability in RAW264.7 cells [24]. Similarly, Ripoll et al. have described that the mouse with lupus nephritis were benefited from the treatment of JAK/STAT pathway blocking [25]. In this study, we found that the phosphorylation of the JAK2 and STAT3 were significantly improved with the upregulation of the AGT. Angiotensinogen (AGT) is involved in the production of angiotensin II (ANG II) which is the main mediator of action of the rennin-angiotensin system (RAS) whereas the RAS mediates the regulation of sodium homeostasis, blood pressure, and inflammation [26]. Also it was found that overexpression of AGT from adipose tissue induced adipose inflammation, and AGT gene silencing could reduce inflammation in cultured adipocytes [27], but it was seldom selected as the candidate that may play critical roles in BPD formation of the infant. In this study, we found that hyperoxic condition can induce the overexpression of the AGT, and subsequently directly or indirectly stimulate the phosphorylation of the Jak2, then activate the Stat3 phosphorylation, whereas the activated jak2/stat3 signal pathway can induce the inflammation, ultimately led to cell damage (Figure 8).

It has been shown that overexpression AGT can over activate the Jak2/Stat3 signaling pathway, lead to inflammation of the cells, and ultimately arrest the proliferation of the cell. Therefore, further studies are needed to study the mechanism of how AGT stimulate the phosphorylation of the Jak2/Stat3 signaling pathway.

In conclusion, the present study demonstrated for the first time that overexpression of Agt inhibits the proliferation of the cells via the Jak2/Stat3 signaling pathway. These results suggest prevention of the BPD may benefit from AGT inhibitors.

## MATERIALS AND METHODS

### Data processing and analysis

#### Microarray data

Microarray data set GSE25286 was downloaded from Gene Expression Omnibus [GEO: GSE25286], including 3 BPD samples and 2 healthy controls [28], The GLP1261 [Mouse430_2] Affymetrix Mouse Genome 430 2.0 Array (Affymetrix, Santa Clara, CA, USA) and the annotation information of probes were used to detect the gene expression.

#### Data normalization and screening of DEGs

The raw data were normalized by using the Robust multichip average(RMA) algorithm in R package affinity following the three steps: background adjustment, quantile normalization, and log 2 transformation. After normalizing the microarray data, we identified the DEGs between the BPD samples and healthy controls by using the Benjamini-Hochberg method, a false discovery rate (FDR) less than 0.05 and an absolute log fold change (|logFC|) greater than 1.5 were set as the significant cutoffs.

#### GO enrichment analysis

Functional enrichment analysis is able to reveal biological functions based on differential expression genes [29]. In this study, web-based DAVID database (Database for Annotation Visualization and Integrated Discovery) was used for Gene Ontology (GO) annotation. The enriched GO terms with P < 0.05 were selected as the significant factors.

#### Construction of interaction network

Proteins usually interact with other proteins to execute functions [30], Therefore, interactors of the most significant DEGs were predicted, including the upregulated DEGs and downregulated DEGs using STRING (Search Tool for the Retrieval of Interacting Genes/Proteins) [31]. Then the protein interaction networks were established. STRING connects major databases and predicts interactions based upon experiments, text mining and sequence homology. Interaction networks from STRING were obtained with a high degree of confidence.

### Cell line and culture conditions

A549 cells were obtained from the Shanghai Institutes for Biological Sciences, Chinese Academy of Sciences (Shanghai, China), and incubated in Roswell Park Memorial Institute(RPMI) medium (GIBCO, USA) containing 10% (v/v) fetal bovine serum (FBS)(GIBCO USA), penicillin and streptomycin were also added with the final concentration of 100 IU/mL, 100 μg/mL, respectively (TaKaRa CHINA). The cells were maintained at 37 °C under humidified 5% CO_2_ in a stationary culture, and were subcultured into two plates, one was in the normoxic control conditions, the other was in the hyperoxic conditions(80% oxygen) for 60h for the further analysis [32].

### Reverse transcription-quantitative polymerase chain reaction (RT-qPCR)

Total RNA was extracted from the sample cells using TRIzol reagent (TaKaRa, China) according to the manufacturer's instructions. The concentration and the purity of total RNA was accessed by the spectrophotometry(GE, USA) according to previous report with modification [33], and the obtained total RNA was stored at -80°C. Then the Total RNA was reversed trancribed into cDNA by the PrimeScript RT Reagent (TaKaRa, Japan). The obtained cDNA acted as the template for RT-qPCR to evaluate of the relative mRNA levels of *FOS, AGT, EDN1, PTGS2, EGR1, SERPINE1,* and *β-actin* which was used as the internal control. The sequences for each primer (Takara, China) are shown in Table 1. The expression levels of genes mentioned above were normalized to *β-*actin and fold-difference in expression level was calculated using comparative C_t_ method.

**Table 1:** Oligonucleotides used as primers for real-time RT-PCR

Gene name	Oligonucleotide sequence	
	Forward	Reverse
*FOS,*	GGGGCAAGGTGGAACAGTTAT	CCGCTTGGAGTGTATCAGTCA
*AGT*	GTGGAGGTCCTCGTCTTCCA	GTTGTAGGATCCCCGAATTTCC
*EDN1*	CTTTGAGGGACCTGAAGCTG	CTGTTGCCTTTGTGGGAAGT
*PTGS2*	ATGCTGACTATGGCTACAAAAGC	TCGGGCAATCATCAGGCAC
*EGR1*	CTGCTCAGTTCGTGCTCACT	CTCCTCGCTCCTCCTCCC
*SERPINE1*	GCGCTGCAGAAAGTGAAGAT	GCGGGCTGAGACTATGACAG
*β-ACTIN*	CTGGAACGGTGAAGGTGACA	AAGGGACTTCCTGTAACAATGCA

### siRNA knockdown experiments

The siRNA sequences targeting AGT was as follows: Forward, 5’- TGTTCCTTGGAAGGACAAGAA-3’ and reverse, 5’- TGTTCCTTGGAAGGACAAGAA-3’. The siRNA were transfected into A549 cell using Lipofectamine ^®^ 2000 (Invitrogen; USA). 2-ml transfection system contain 12ul Lipofectamine ^®^ 2000 and siRNA. After 24h of transfection, the cells were replaced by RPMI-1640 basal medium for 24 h and were then processed for the further study.

### Elisa analysis

The A549 cells were treated with or without ANG II (10^-7^M) and Valsartan (10^-6^M) under the condition of the hyperoxic or room air, respectively. The supernatant of the A549 cells with different treatments were collected from the 6 wells-culture plates. The levels of pro-inflammatory cytokines (IL-1β and IL-6) in culture medium were assessed by commercially available ELISA Kits (R&D Systems) according to the manufacturer's instructions.

### Western blot analysis

Western blot was carried out as described before [34, 35], briefly, aliquots of lysed cells were loaded into SDS-PAGE in gels and transferred to PVDF. After blocking in 5% BSA/TBS/0.1% Tween-20 for 2h at room temperature, the membranes were immunoblotted with anti-p-JAK2, anti-p-STAT3, anti-JAK2, anti-STAT3, anti-β-actin, anti-GAPDH (All antibodies were purchased from Abcam (Abcam, Cambridge, UK)) in TBS/0.1% Tween-20 overnight at 4°C. After washing with TBS/0.1% Tween-20, membranes were incubated with secondary antibody conjugated with HRP in TBS/0.1% Tween-20. The bands were visualized with High-sig ECL Western Blotting system (Tanon, China).

### Cell viability assay

Approximately 1×10^4^ A549 cells were transferred to each well of the 96-well plate, and After incubating in normoxic (control) or hyperoxic conditions, and with or without treatment of ANG II and Valsartan, the cell viability was evaluated by measuring the mitochondrial-dependent reduction of colorless 3-(4,5-dimethylthiazol-2-yl)-2,5-diphenyltetrazolium bromide (MTT; TaKaRa, China) to a blue-colored formazan that was dissolved in dimethyl sulfoxide as describe elsewhere [36] and the absorbance of each sample was spectrophotometrically measured at 550 nm with a Bio Kinetics reader (BIO-TEK USA).

### Statistical analysis

All data are presented as the means ± standard deviation (SD). Comparisons among groups were performed by one-way ANOVA using SPSS 17.0 software (SPSS, Inc., Chicago, IL, USA). All experiments were repeated three times. P<0.05 was considered to indicate a statistically significant difference.
